# Spillover effects from invasive *Acacia* alter the plant–pollinator networks and seed production of native plants

**DOI:** 10.1098/rspb.2023.2941

**Published:** 2024-04-10

**Authors:** Maisie F. Brett, Paula Strauss, Kurt van Wyk, Ian P. Vaughan, Jane Memmott

**Affiliations:** ^1^ School of Biological Sciences, University of Bristol, Bristol BS8 1TQ, UK; ^2^ Grootbos Foundation, Gansbaai 7220, Western Cape, South Africa; ^3^ Cardiff School of Biosciences, Cardiff University, Cardiff CF10 3AX, UK

**Keywords:** pollinators, networks, invasive plant, fynbos, spillover

## Abstract

Invasive flowering plants can disrupt plant–pollinator networks. This is well documented where invasives occur amongst native plants; however, the potential for ‘spillover’ effects of invasives that form stands in adjacent habitats are less well understood. Here we quantify the impact of two invasive Australian species, *Acacia saligna* and *Acacia longifolia*, on the plant–pollinator networks in fynbos habitats in South Africa. We compared networks from replicate 1 ha plots of native vegetation (*n* = 21) that were subjected to three treatments: (1) at least 400 m from flowering *Acacia;* (2) adjacent to flowering *Acacia*, or (3) adjacent to flowering *Acacia* where all *Acacia* flowers were manually removed. We found that native flowers adjacent to stands of flowering *Acacia* received significantly more insect visits, especially from beetles and *Apis mellifera capensis*, and that visitation was more generalized. We also recorded visitation to, and the seed set of, three native flowering species and found that two received more insect visits, but produced fewer seeds, when adjacent to flowering *Acacia*. Our research shows that ‘spillover’ effects of invasive *Acacia* can lead to significant changes in visitation and seed production of native co-flowering species in neighbouring habitats—a factor to be considered when managing invaded landscapes.

## 1. Introduction

Invasive plant species that rely on pollination by animals for successful fertilization must become integrated into local plant–pollinator networks in order to become established in their new range [1]. These invasive species can disrupt local ecosystems by altering the structure and function of local plant–pollinator networks [2,3]. For example, the reproductive success of co-flowering native plants can be negatively affected, either via a preference by local pollinators for invasive plants resulting in fewer visits to native plants [2,4–6], or via the deposition of invasive pollen on native stigmas [6]. Both mechanisms have the potential to affect the reproduction of co-flowering species [7]. Whilst the majority of studies on invasive plant species focus on their impact on just a single native species (e.g. [4,8,9]) or a few (e.g. [6]), the alternative approach of investigating the impact on all co-flowering species can yield important insights into the overall effect on the community [10].

Invasive plant species can establish themselves by growing amongst native species in their new range, i.e. becoming completely integrated into the native community. Alternatively they can form dense single-species stands of vegetation that exclude most, or all, native plants. These dense stands raise the possibility of ‘spillover’ effects. A spillover effect is the net movement of species over the boundary of one habitat into another and such effects are widely reported in, for example, predator–prey [11] and host–parasite [12] interactions, in marine reserves and fishing grounds [13], and in urban–rural intersections [14]. They are also reported regarding the movement of pollinators from semi-natural habitats into crops [15], and the aim of this study is to test for spillover effects in an invasive plant context. Spillover effects of bordering cropland upon native plants are well described, causing, for example, pollinator dilution [16,17] or increasing numbers of agriculturally subsidized natural enemies [18–20]. However, there is a dearth of such literature regarding spillover effects of invasive plants, and both agricultural and invasive contexts lack community-level plant–pollinator network studies ([21], but see [22]). Thus, do pollinators visiting dense stands of flowering invasive plants spill over into native habitats and impact adjacent, uninvaded plant–pollinator communities, i.e. impact at a distance? Given the global prevalence of invasive flowering plants that can establish as single-species stands (e.g. *Rhodedenron ponticum*, *Impatiens glandulifera* and *Lythrum salicaria*), understanding their impacts on the pollination of adjacent habitats through possible spillover effects is important.

**Figure 1.**
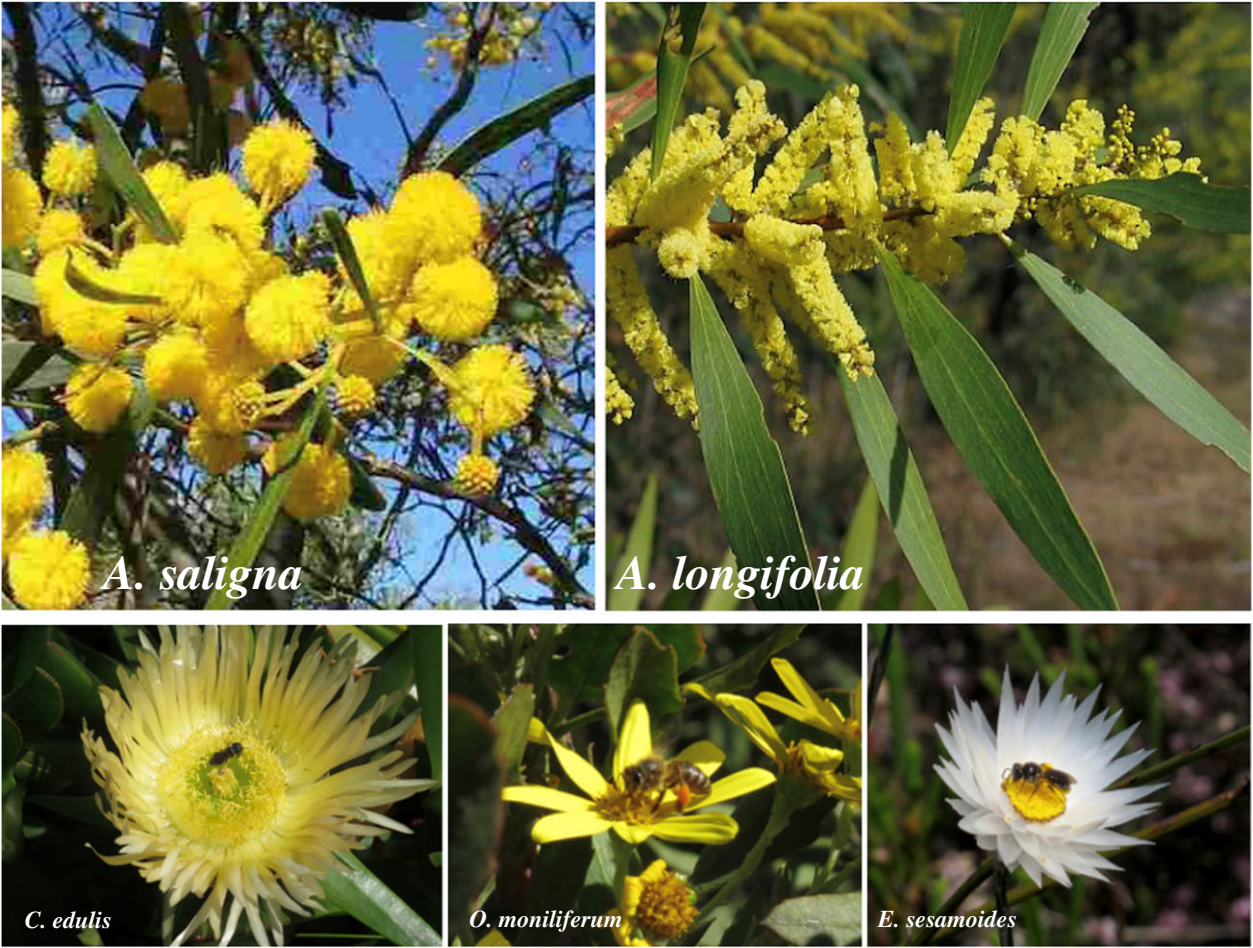
*Acacia saligna* (left) and *Acacia longifolia* (right), and the three focal co-flowering fynbos species *Carpobrotus edulis, Osteospermum moniliferum* and *Edmondia sesamoides*.

First recorded in South Africa in 1883, invasive *Acacia saligna* and *Acacia longifolia* (hereafter referred to collectively as ‘*Acacia*’) are of particular concern to the economy and biodiversity of the country [23,24]. Both species form dense stands of woody shrubs in native fynbos vegetation and have large yellow inflorescences (figure 1). Fynbos habitats characterize the Cape Floristic Region and are renowned for their diversity and endemism of plant species [24]. Out of all invasive plants growing in Mediterranean habitats, Australian *Acacia* species in South Africa account for the largest declines in native plant species richness, owing to suppression of co-occurring plants [25] at which they are effective [26], thereby forming single-species stands. Both *Acacia* species receive visits from predominantly generalist flower-visiting insects such as beetles and honeybees, and flower from July to October (winter to early spring), thus coinciding with the flowering of a majority of the native fynbos species [6,24]. Indirect affects of *Acacia*, such as through changes in native plant pollination, are less well understood.

There are two objectives to our research: first, to quantify the ‘spillover’ effect of dense stands of *Acacia* on the adjacent plant–pollinator networks, which are a powerful tool when trying to understand how invasive plants affect entire communities of interacting species (3); second, to quantify the impact of the stands of *Acacia* on the seed set of native plants, thus providing an estimate of their impact on recruitment into the next generation of plants.

## 2. Methods

We tested the effects of *Acacia* on pollinator behaviour using two complementary methods. First, we compared plant–pollinator networks from replicate field plots that were distant or adjacent to *Acacia* stands*,* to investigate changes in plant–pollinator network structure in response to proximity to flowering *Acacia*. Second, we compared the insect visits to, and seed production of, three native flowering plants in plots that were distant or adjacent to *Acacia* stands. Observing significant changes in plant–pollinator network structure, or changes in seed production at the ‘invaded’ plots, would indicate a spillover effect caused by changes in the behaviour of flower visitors due to proximity to invasive *Acacia* flowers.

### (a) Plot selection

The study took place in the Overstrand municipality, in the Western Cape of South Africa (34°32'02″ S, 19°25'54″ E). Of the several species-rich fynbos vegetation types present in the area, three were selected for investigation: Overberg Sandstone, Elim Ferricrete and Agulhas Sand fynbos [18]. Our twenty-one 1 ha field plots were at least 500 m apart to minimize pollinator dispersal among the plots. The three plot types are listed below:

1) *Distant plots*: Fynbos habitat with no invasive *Acacia* species within 400 m (the greatest distance feasible within the landscape).
2) *Invaded plots:* Fynbos habitat adjacent to an area infested by flowering *Acacia* vegetation.
3) *Invaded plots with flowers removed*: Fynbos habitat adjacent to a stand of invasive *Acacia*, but the flowering branches of *Acacia* were removed, leaving just the vegetative structure remaining. Removing just the flowering branches allows the impact of *Acacia's* floral resources to be separated from other impacts, such as the competitive impact on native vegetation (e.g. [21]).

**Figure 2.**
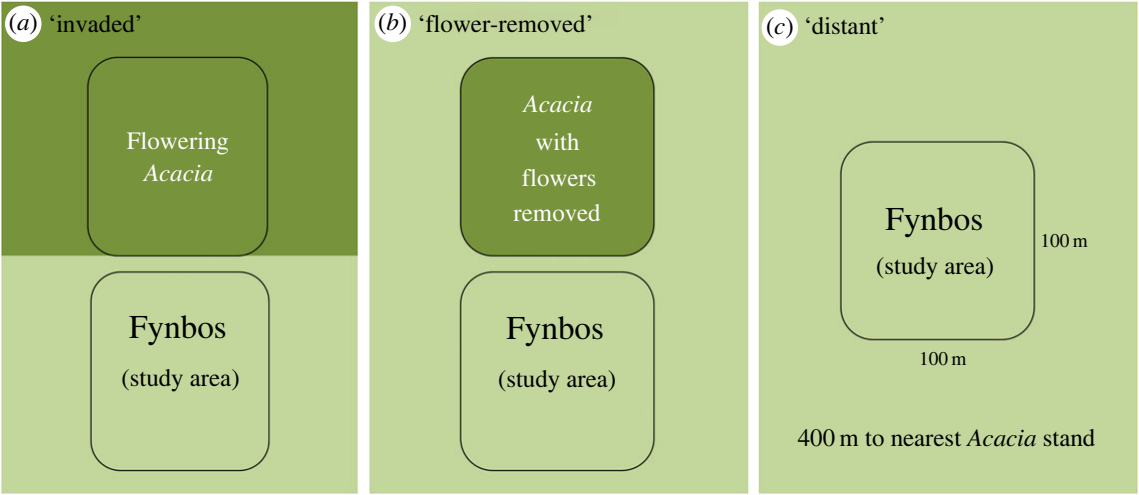
The three types of field plot. (*a*) Invaded: fynbos habitat adjacent to flowering *Acacia*; (*b*) flower-removed: fynbos habitat adjacent to a smaller stand of *Acacia* from which flowering parts had been removed; (*c*) distant: fynbos habitat with no *Acacia* present for 400 m in any direction.

**Figure 3.**
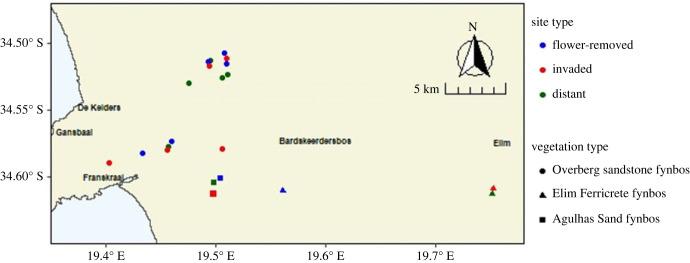
The distribution of the 21 study plots within the Overstrand municipality of the Western Cape, South Africa, with shape indicating vegetation type and colour indicating treatment for each plot. Blocked sites were not always adjacent, owing to the multiple criteria used in site selection.

Together, the three plot types enable us to quantify the impact of the *Acacia* flowers on the adjacent area, thus determining whether a spillover effect occurs. A diagram of the three treatments is shown in figure 2, a map of sites in figure 3, and illustrations of the surrounding landscape composition for each plot in electronic supplementary material, figure S1. The distance from ‘distant’ plots to nearest flowering *Acacia* was 398–1931 m (mean 1081 ± 591 m). At invaded and flower-removed plots, the nearest investigated native plants to *Acacia* ranged from 0.5–195 m (mean = 86 ± 59 m) distant. With regard to the ‘distant’ plots, 400 m is within the flying range of larger pollinators such as carpenter bees (*Xylocopa*) and the Cape honeybee (*Apis mellifera capensis*) [27,28]. Therefore, the experiment investigates the effect of distancing from *Acacia*, rather than total isolation, within a generally invaded landscape.

To quantify the relative isolation of the plots from the nearest stands of *Acacia*, a distance metric weighted by the area of the nearest *Acacia* stand from the outer edge of each plot was calculated using the connectivity index described in [29]. *Acacia* flower removal was carried out two weeks prior to the start of the flowering season (i.e. with trees in bud), to allow the vegetation to recover from initial disturbance. The two *Acacia* species varied in abundance between sites; however, their floral density was comparable (electronic supplementary material, table S1) and can be said to have similar effects on native pollinators. To minimize the impact of variation in floral community among the three treatments, the plots were mapped in seven triplets containing one of each treatment, using the following information: (1) fynbos vegetation type; (2) habitat age, i.e. time since last wildfire; (3) mean vegetation height; (4) floral assemblage, and (5) altitude. Locations and detailed information for all plots including *Acacia* isolation metrics are provided in electronic supplementary material, table S1; vegetation types were allocated per Rebelo *et al.* [24].

### (b) Identifying the impact of *Acacia saligna* and *Acacia longifolia* on plant–pollinator networks

Plant–pollinator visitation data were collected from the 21 plots between late July and mid-October in 2019. This time encompasses the peak flowering time of the *Acacia* and native fynbos plants. Each plot was surveyed four times, leading to a total of 84 surveys. Surveys took place between 09.00 and 17.00. Wind speed and temperature were measured with a hand-held anemometer (Kestrel 3000, Kestrel Meters, Boothwyn, USA). Surveys were only conducted when there was less than 50% cloud cover, no rain, wind speed below 10 m s^−1^ and temperatures above 21°C. At each plot, consecutive surveys were carried out at a different time of day, rotating through 09.00–12.00,12.00–14.00 and 14.00–17.00 to account for temporal variation in insect behaviour and pollen/nectar provision, as in Gibson *et al.* [6].

During each survey, the flowering plants in each plot were sampled using four randomly placed 50 m transects, recording all flowering species and the number of floral units for each species within a 2 m × 2 m quadrat, placed every 10 m along the transect. A floral unit was defined as that which a medium-sized bee has to fly, rather than walk, between to access [30]. Floral data from the 20 quadrats (4 transects, each with 5 quadrats) were pooled for each plot for each survey. These data were used to quantify the floral species richness and abundance in the plots. Plant–pollinator interactions were recorded during a 1 h haphazard walk within each 1 ha plot. A plant–pollinator interaction is defined as an insect making contact with the reproductive parts of a flower. For each interaction observed, the timer was paused, the insect and flower species were recorded, and when the insect visitor species was unknown, a specimen was collected for later identification (94% of observations). These specimens are stored in the Grootbos Foundation entomological collection.

To identify any overlap in insect species visiting invasive *Acacia* and those visiting native flora, an additional 20 min timed observation walking along the *Acacia*–fynbos habitat boundary and recording all visits to the flowering *Acacia* next to each ‘invaded’ plot were carried out. Density of *Acacia* invasion precluded carrying out these surveys within the *Acacia* stand itself. Given the abundance of inflorescences on *Acacia*, a correlation between tree size and number of inflorescences was used to provide an estimate of *Acacia* floral density per plot. Thus, all *Acacia* plants within a 10 m × 10 m quadrat at each invaded site were counted and their circumference was measured at chest height. The regression equations from these data provide an estimate for the floral density of *A. longifolia* and *A. saligna* adjacent to each plot (see electronic supplementary material, tables S1 and S2).

Sampling completeness was compared between the three treatments by generating Chao values, using the vegan package [31] in R (version 3.6.2.; [32]). Values were generated for insect and plant species richness, and the richness of plant–insect interactions for each plot. The following indices were then calculated for the plant–pollinator network at each plot, using the bipartite package in R [33,34]: (a) links per species (the mean number of interactions per species, a measure of specialization); (b) interaction evenness (a measure of species functional diversity based on links per species—see [33,34]); (c) weighted nestedness (the degree of nestedness of a network, accounting for species abundances); (d) mean number of insect visits per floral unit; and (e) plant and insect species richness. We selected these five metrics as together they provide a good summary of network functionality, through which to detect ecological change affecting native plant pollination. These indices were then treated as response variables and tested against the fixed variables of ‘treatment’ (distant, invaded, flower-removed), including random effect of ‘vegetation type’ (Overberg Sandstone, Elim Ferricrete, Agulhas Sand), in generalized linear mixed models (GLMMs), using the glmmTMB package [35] in R. The choice of link function for each model is shown in table 1 under the heading ‘model type’ and these functions were selected based on the data distributions for each variable.

**Table 1.** Mean values **±** 1 s.e. of network response variables, and results of generalized linear mixed models (GLMMs) and Tukey *post hoc* tests between distant (P), flower-removed (F) and nvaded (I) plots. Fixed variables were 'plot type', with 'vegetation type' included in models as a random effect. Significant *p*-values are shown in bold type. *C. edulis*, *Carpobrotus edulis*; *O. moniliferum*, *Osteospermum moniliferum*; *E. sesamoides*, *Edmondia sesamoides*.

variable	mean + 1 s.e. across all plots, surveys pooled		model type	effect of treatment			Tukey *post hoc* tests	
	distant	invaded		*F*-value	*p*-value	d.f.	direction	*p*-value
*visitor species richness*								
all insects	25.89 ± 0.67	25.11 ± 0.54	negative binomial	4.255	**0**.**037**	1	P > I	**<0**.**001**
*C. edulis*	25.72 ± 0.12	30.16 ± 0.34					P < I	**<0**.**001**
*E. sesamoides*	23.37 ± 0.18	19.41 ± 0.51					P > I	**<0**.**001**
*O. moniliferum*	28.51 ± 1.01	24.81 ± 0.78					P > I	**<0**.**001**
Coleoptera	11.33 ± 0.44	9.333 ± 0.46	Poisson	1.751	0.210	1	—	—
Hymenoptera	5.44 ± 0.31	6.33 ± 0.54	Poisson	0.547	0.474	1	—	—
Diptera	3.00 ± 0.40	3.56 ± 0.28	negative binomial	0.168	0.688	1	—	—
*no. visits per floral unit*								
all insects	3.79 ± 0.02	3.12 ± 0.02	negative binomial	9.108	**0**.**003**	1	P > I	**<0**.**001**
*C. edulis*	9.51 ± 0.47	5.88 ± 0.59					P > I	**<0**.**001**
*E. sesamoides*	1.46 ± 0.11	1.28 ± 0.06					P > I	0.909
*O. moniliferum*	0.70 ± 0.10	0.66 ± 0.09					P > I	0.577
Coleoptera	2.58 ± 0.34	0.78 ± 0.06	Gaussian	36.530	**>0**.**001**	1	P < I	**<0**.**001**
*C. edulis*	6.22 ± 0.23	1.78 ± 0.09					P > I	**<0**.**001**
*E. sesamoides*	0.64 ± 0.56	0.60 ± 0.05					P > I	1.000
*O. moniliferum*	0.51 ± 0.11	0.36 ± 0.08					P > I	1.000
Hymenoptera	0.27 ± 0.03	0.40 ± 0.03	Gaussian	2.283	0.157	1	—	—
Diptera	0.09 ± 0.02	0.13 ± 0.01	Gaussian	0.306	0.588	1	—	—
*seed set*								
mean seed set per floral unit	88.78 ± 0.65	78.53 ± 0.62	begative		0.063		P > I	0.633
*C. edulis*	1211.65 ± 11.49	1206.26 ± 8.60	binomial				P > I	0.997
*E. sesamoides*	105.79 ± 0.47	97.29 ± 0.85					P > I	**0**.**002**
*O. moniliferum*	1.95 ± 0.004	1.855 ± 0.004					P > I	**<0**.**001**

While variation in native floral assemblage among the three fynbos vegetation types was significant (see electronic supplementary material, analysis S1), native floral assemblage did not differ significantly among the three treatments, nor did floral species richness (see Results). Therefore, floral assemblage is not an obvious confounding variable, and vegetation type was included in the GLMMs as a random effect. Where GLMM results were significant, *post hoc* Tukey tests were performed to assess differences in network indices between treatments and among the most common visitor orders (Coleoptera, Hymenoptera and Diptera) using the multcomp package [36].

### (c) Quantifying the effects of *Acacia* on insect visits and seed production of three native flowering species

Three native co-flowering plant species were chosen to quantify the effect of *Acacia* on native seed production, using characteristics that were predicted to increase overlap in pollinators with *Acacia*. These were *Carpobrotus edulis* (Azoaecae), *Osteospermum moniliferum* (Asteraceae) and *Edmondia sesamoides* (Asteraceae), illustrated in figure 1. It has been shown that fynbos flowering species that have comparable floral symmetry, clustering and colour to *Acacia* have a greater overlap in flower visitors [5,6]. Thus, two of the focal species had yellow flowers (*C. edulis* and *O. moniliferum*) and the third had a yellow centre (*E. sesamoides*), and all three were radially symmetrical. For each focal species, populations were identified within three of the distant and three of the invaded plots adjacent to flowering *Acacia,* all within Overberg Sandstone habitats. Logistical constraints precluded using the flower-removed plots for this experiment.

To measure insect visitation, three fixed-point surveys of 20–30 min were carried out at each plot for each focal species, each on a separate day and rotating time period as previously described. Each focal species received a total of 180–220 min of observation, and insect visitation data from the three surveys were pooled for analysis for each focal species. The floral abundance of the focal plants in each plot was recorded to calculate the insect visitation frequency per focal species (i.e. the number of pollinator visits per floral unit).

To quantify the impact of *Acacia* on seed production, equal numbers of mature seed heads were collected from the distant and invaded plots. Seed heads collected from invaded plots were under 100 m from flowering *Acacia*. Thus, 140 *O. moniliferum*, 60 *E. sesamoides* and 30 *C. edulis* seed heads were collected for each treatment. The impact of *Acacia* on native plant recruitment may be lessened in species that can self-pollinate. Therefore, the extent of self-pollination in each species was measured by comparing seed set in open and pollinator-excluded flowers. Pollinators were excluded by placing mesh bags over 10–20 randomly selected flower heads of each species, the seed heads allowed to mature within the bags, and the individual seeds counted manually.

The number of insect visits per floral unit, insect species richness and seed set (the total seed produced per focal flowering species) were treated as response variables and tested against the fixed variables of plot type and focal species using GLMMs. In the case of strongly skewed distributions (such as seed count data), negative binomial type models were used. Where results were significant, *post hoc* Tukey tests were used to assess differences among the most common insect visitor orders (Hymenoptera, Coleoptera and Diptera).

## 3. Results

### (a) Identifying the impact of *Acacia saligna* and *Acacia longifolia* on plant–pollinator networks

Isolation metrics differed significantly between ‘distant’ and ‘invaded/removed’ sites, reflecting a real difference in the proximity of the treatments to *Acacia*. The results of this analysis are provided in electronic supplementary material, analysis S2 and table S1. In total, 7188 flower–visitor interactions were recorded in the 21 plots, with 660 insect morphospecies visiting 168 plant species. Ninety-one per cent of visits were from Hymenoptera, Coleoptera and Diptera (39, 38 and 12% respectively). The species richness for plants was 95.95% of the predicted true species richness from Chao-generated values (322 ± 7.48) and the insect richness was 54.23% of predicted true species richness (1218 ± 87.14). The latter result was relatively consistent among invaded, distant and flower-removed plots (57, 52 and 48% respectively). Chao-generated values for plant–insect interaction richness were consistent among treatments—at 49.66 (± 2.20) for invaded sites, 46.93 (± 2.88) for flower-removed sites and 42.60 (± 3.44) for distant sites. Thus, it is legitimate to compare the networks among the three plot types without need for data rarefaction. The 21 plant–pollinator networks each contained 84 to 123 insect morphospecies (mean = 110), which visited 25 to 55 plant species (mean = 39). Further information on Chao estimates, observed plant and insect taxa, and an example of a triplet of visitation networks are provided in electronic supplementary material, figure S2 and tables S3–S5.

#### (i) Impact of invasive *Acacia* on plant–pollinator network structure

**Figure 4.**
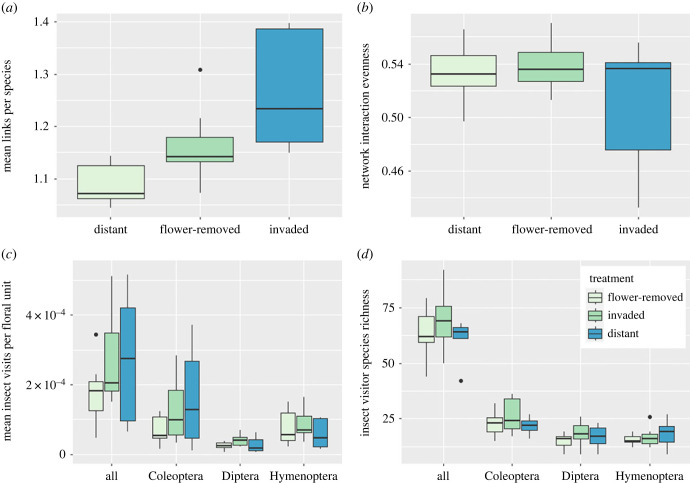
Plots showing the median and inter-quartile range at invaded, distant and flower-removed plots for (*a*) links per species, (*b*) lnteraction evenness, (*c*) lnsect visits per floral unit by all insects, Coleoptera, Diptera and Hymenoptera, and (*d*) insect visitor species richness for all insects, Coleoptera, Diptera and Hymenoptera.

The mean links per species were significantly higher at invaded plots, compared with distant plots and flower-removed plots (table 1, figure 4*a*). After separating plant and insect data, the mean links per plant species did not differ amongst treatments; however, mean links per insect taxa remained significantly higher at invaded plots compared with distant plots (table 1). Thus, overall, plant species are not being visited by more insect morphospecies, but the insect morphospecies are visiting more plant species. An non-metric multi-dimensional scaling analysis of Coleoptera assemblages weighted by links per species showed that Coleoptera taxa visiting more than one plant taxon were more frequent at invaded plots (electronic supplementary material, analysis S1). There were no significant differences in interaction evenness or weighted nestedness amongst treatments (table 1, figure 4*b*).

There were significantly more insect visits per floral unit per survey at the invaded plots compared with flower-removed and distant plots, but no difference between flower-removed plots and distant plots (table 1, figure 4*c*). Coleoptera made significantly more visits per survey to plants at invaded plots compared with distant plots and flower-removed plots, but there was no significant difference in the number of insect visits among treatments for Hymenoptera or Diptera. Insect visitor species richness per survey did not differ significantly among treatments (table 1, figure 4*d*).

At the invaded plots, the density of adjacent *Acacia* flowers was on average 11.6 times that of fynbos flowers. However, the mean number of insect visits per floral unit per survey was 13.3 times greater for fynbos species than for *Acacia*. Importantly, all insect visitors recorded visiting *Acacia* flowers were also recorded visiting fynbos species, including 11 of the 15 most abundant insect visitors to fynbos plants. Inversely, 34% of all visits to fynbos flowers were made by insects also recorded on *Acacia*. Of the 59 insect taxa visiting *Acacia* flowers, Hymenoptera made 50.4% of visits, Coleoptera 33.6% and Diptera 1.4%, with the remainder being made by four other orders. *Apis mellifera capensis* contributed 27.4% of all visits to *Acacia* and 16.6% of visits to fynbos plants overall. See electronic supplementary material, table S2 for a complete list of *Acacia* insect visitor species.

### (b) Quantifying the effects of *Acacia* on insect visits and seed production of three native flowering species

#### (i) Impact of *Acacia* on insect visitation

**Figure 5.**
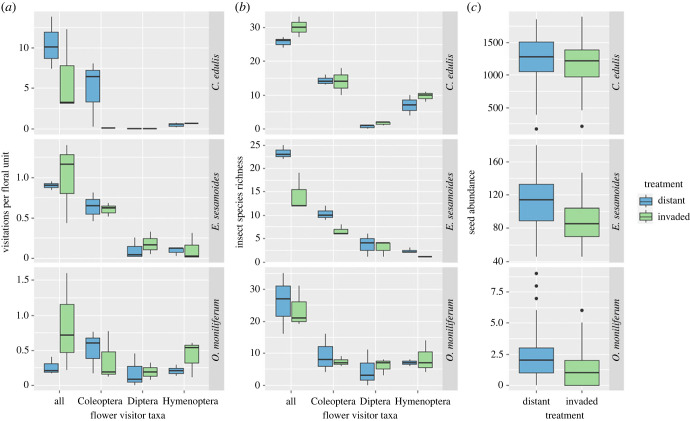
Boxplots showing differences between invaded and distant plots for the three focal species: (*a*) visitation per floral unit for different insect orders, (*b*) visitor species richness per plot for different insect orders, and (*c*) mean number of seed produced per flower, for *Carpobrotus* *edulis* (top)*, Edmondia sesamoides* (centre) and *Osteospermum moniliferum* (bottom). Note the different *y*-axis scale values for each species.

Visits by Coleoptera to *C. edulis* were significantly reduced at invaded plots (table 2, figure 5*a*). There were no significant differences in the number of visits made by Hymenoptera and Diptera in the invaded and distant plots to any of the three focal plant species. Insect visitor species richness was significantly higher at distant plots than at invaded plots for *E. sesamoides* and *O. moniliferum* (table 2). In contrast, for *C. edulis,* overall visitor species richness was significantly lower at distant plots (table 2, figure 5*b*). For insect visitor species richness for the three focal plant species, see electronic supplementary material, table S6.

**Table 2.** Mean values **±** 1 s.e. of response variables, and results of generalized linear mixed models (GLMMs) and Tukey *post hoc* tests for differences between distant (D) and invaded (I) plots for focal species. Fixed variables were plot type (distant or invaded) and focal plant species. Significant *p*-values are shown in bold type.

variable/taxon	mean ± 1 s.e. across all plots, surveys pooled			model type	effect of treatment			Tukey *post hoc* tests	
	distant (D)	flower-removed (F)	invaded (I)		*F*-value	*p*-value	d.f.	direction	*p*-value
*Insect species richness*									
all insects	68.14 ± 4.69	74.85 ± 5.46	67.14 ± 4.03	Gaussian	0.323	0.730	2	—	—
Coleoptera	31.57 ± 1.36	25.43 ± 0.37	21.71 ± 0.21	negative binomial	0.421	0.666	2	—	—
Hymenoptera	16.57 ± 2.13	16.43 ± 1.82	16.57 ± 2.29	Poisson	0.002	0.999	2	—	—
Diptera	18.57 ± 2.39	19.00 ± 2.62	16.38 ± 1.71	Gaussian	0.367	0.699	2	—	—
*No. visits per floral unit*									
all insects	0.061 ± 0.008	0.075 ± 0.011	0.143 ± 0.038	Gaussian	3.417	0.058	2	—	—
Coleoptera	0.016 ± 0.002	0.003 ± 0.007	0.074 ± 0.017	Poisson	6.819	**0**.**007**	2	I > P	**0.005**
								P > C	0.264
								I > C	**0.001**
Hymenoptera	0.02 ± 0.003	0.023 ± 0.003	0.07 ± 0.04	Gaussian	1.874	0.186	2	—	—
*Apis mellifera capensis*	0.002 ± 0.001	0.001 ± 0.003	0.004 ± 0.003	Gaussian	1.324	0.293	2	—	—
Diptera	0.01 ± 0.001	0.01 ± 0.001	0.01 ± 0.001	Gaussian	1.571	0.248	2	—	—
interaction evenness	0.53 ± 0.01	0.54 ± 0.01	0.51 ± 0.02	Gaussian	1.835	0.192	2	—	—
weighted nestedness	0.45 ± 0.03	0.46 ± 0.05	0.45 ± 0.04	Gaussian	1.5870	0.2351	2	—	—
links per species	1.07 ± 0.01	1.15 ± 0.03	1.23 ± 0.05	Gaussian	6.665	**0**.**011**	2	I > P	**<0.001**
								P < C	0.054
								I > C	**0.045**
links per insect species	2.858 ± 0.03	3.008 ± 0.03	4.078 ± 0.06	Gaussian	3.964	**0**.**047**	2	I > P	**0.021**
								P < C	0.721
								I > C	0.934
links per plant species	12.465 ± 0.22	13.308 ± 0.24	11.281 ± 0.12	Gaussian	0.302	0.744	2	—	0.934

#### (ii) Impact of *Acacia* on seed set

The mean seed set was significantly lower at invaded plots than at distant plots for *E. sesamoides* and for *O. moniliferum*; but there was no significant difference in seed set by *C. edulis* between distant and invaded plots (table 2, figure 5*c*). Regarding the ability of the three species to self-pollinate, *O. moniliferum* flowers fitted with exclusion bags (*n* = 18) produced a mean of 0.10 ± 0.001 seeds, while the open pollinated flowers had mean of 1.94 ± 0.004 seeds (*n* = 840 seed heads). *Carpobrotus edulis* flowers with exclusion bags (*n* = 10) produced 0 seeds, whilst open pollinated flowers had a mean of 1209.03 ± 9.64 seeds (*n* = 60 seed heads). In contrast, *E. sesamoides* with exclusion bags (*n* = 11) produced a mean of 62.3 ± 0.12 seeds, whilst open pollinated flowers had a mean of 101.65 ± 0.47 seeds (*n* = 120 seed heads), indicating some level of self-compatibility. Therefore, although significant changes in seed production were observed for this species, *E. sesamoides* may be somewhat buffered from the population-level effects of *Acacia* invasion.

## 4. Discussion

Invasive *Acacia* species are known to affect many aspects of fynbos ecology in the Western Cape, and our study reports three new effects—that they alter the structure of native plant–pollinator networks, that they affect the reproduction of co-flowering plants through reduced seed set, and that these changes occur in areas adjacent to *Acacia* invasion, i.e. there is a local spillover effect. Our work also presents the first comprehensive plant–pollinator networks for three fynbos habitat types. There is a dearth of community-level pollination studies in fynbos habitats [6,11,37], which are needed to understand how invasive plant species interact with threatened and diverse fynbos communities. In what follows, the limitations of our study are discussed and results are considered in the context of the wider literature.

### (a) Limitations

There are two main limitations to our approach. First, our focal species experiment on seed set was limited by the relatively small number of plots per treatment (*n* = 3) and the small number of selected plant species. The three plant species were selected as ones likely to be affected by *Acacia*, owing to likely sharing of pollinators; a larger selection would however provide a better measure of the average impact of *Acacia* on native plants. Nevertheless, our results show that *Acacia* can negatively affect seed set in native plant species. Secondly, the minimum 398 m distance between flowering *Acacia* stands and ‘distant’ treatments was sufficient to detect an effect on network metrics; however, many locally recorded pollinators can disperse farther than this (e.g. [28,38–40]). This may partially explain the overlap in flower visitor assemblages among treatments and plots. However, the contrast between treatments was sufficient to detect a general spillover effect from the *Acacia* to adjacent plots within a generally invaded landscape. Future studies that incorporate a gradient of sites with *Acacia* present at varying distances from the study plots would allow the absolute (rather than relative) size of this effect to be measured. This study was carried out during the *Acacia* flowering season (from July to October) and future work exploring the impacts of *Acacia* on fynbos habitats during other months and across multiple years may reveal knock-on effects that last beyond a single flowering season. Variation in the temporal impact of invasives, and indeed crops, on neighbouring plant communities has been previously highlighted as a subject warranting attention [41].

### (b) The impact of *Acacia* on plant–pollinator networks

Given that the increase in mean links per species at invaded plots was observed for insect species but not for plant species, the change in network structure is caused by a change in abundance of *generalist insect visitors* rather than in abundance of plants with generalist traits at invaded plots. In other words, at *Acacia*-invaded plots, the mean number of plant species visited by each insect increased, but plant species received a similar number of insect visitors regardless of *Acacia* presence. Comparisons of insect assemblages between treatments revealed that invaded plots contained greater numbers of generalist Coleoptera. A likely explanation for this is that invasive flowering plants tend to attract generalist pollinators [2,20]. The scale of the *Acacia* floral displays, which are obvious from a considerable distance, may provide a significant draw to generalist taxa, which then ‘spills over’ into the adjacent study plot, causing a shift in local insect assemblage, leading to generalization of plant–pollinator networks. Given the low but consistent sampling completeness at the sites, the increase in links per species can be explained by the increase in overall visits by Coleoptera taxa to invaded plots, as the richness of Coleoptera remained unchanged across treatments. Increased links per species in plant–pollinator networks have previously been recorded in response to plant invasions [29] and habitat disturbance [42].

Unexpectedly, insect visits per floral unit to *Acacia* were significantly fewer than to focal fynbos species, indicating that, generally, insects prefer to visit fynbos flowers over *Acacia*. However, given that we estimated density of *Acacia* flowers to be on average over ten times that of Fynbos flowers at invaded plots, they would still provide a significant draw to pollinators and therefore impact visitation to local plant species. The observed increase in visits to flowers at invaded plots compared with other plots initially suggests a facilitative, or ‘magnet’ effect from *Acacia* (e.g. [42]). However, an increase in visits from insects does not necessarily lead to increased reproductive success for plants; indeed, we observed reduced seed set in two of the focal plant species. There are two possible explanations for this. One is that flower visitors vary in their effectiveness as pollinators; therefore an increase in visits by less mobile insects, such as Coleoptera [38,39], may not imply a pollination advantage. Another explanation is that the stigmas of native plants can be inundated with invasive pollen through heterospecific pollen transfer ([2], though see [43]). Importantly, neither *Acacia* species produces floral nectar [44–46]; therefore nectar-dependent insects visiting *Acacia* for pollen would need to supplement their visits to *Acacia* with nectar-producing fynbos plants in the local area. This would increase the potential for transfer of invasive pollen to native stigmas and may explain an increase in flower visits to nectar-producing native plants adjacent to *Acacia*. For example, nectar-dependent *A. mellifera capensis* was the primary visitor to both *Acacia* species, making up 27% of visits overall, but also the primary visitor to 29 native plant species, increasing the likelihood of visitor overlap between *Acacia* and native flora and thus changes to plant–pollinator network structures. A significant increase in flower visits at invaded plots was recorded for Coleoptera, but not for Diptera, Hymenoptera, or *A.mellifera capensis* specifically. Aside from visits from *A. mellifera capensis*, Coleoptera species contributed the most visits to *Acacia* (33.6%); therefore the increased frequency of visits by Coleoptera at invaded plots may be attributed to the presence of *Acacia* floral resources nearby.

Neither insect nor plant species richness differed among the three treatments; this implies that species richness is maintained despite network structure changes due to proximity to *Acacia* stands. Similar results have been reported in food webs (e.g. [47,48]) and reflect the plasticity or ‘re-wiring’ of interactions that a variety of ecological networks can display in response to ecological disturbance (e.g. [49]). The differences found between ‘flower-removed’ and ‘invaded’ treatments highlights the separate effects of *Acacia* vegetation and floral displays altering local flower-visitation networks, as described by [36]. These separate effects would have been overlooked in a study comparing only invaded and distant plots, highlighting the utility of manipulative field-based approaches to understanding underlying mechanisms in ecological network studies [50].

### (c) The effects of *Acacia* on insect visits and seed production of three native flowering species

The mean number of insect visits to *E. sesamoides* and *O. moniliferum* remained unchanged at invaded plots, and so a change in the *quality* of visitation to flowers is likely to be an underlying cause of the reduced seed production observed in these species. The reduced species richness of insect visitors at invaded plots for *O. moniliferum* and *E. sesamoides* may have reduced the quality of flower visits, owing to a potential loss of diversity in visitor functional groups or efficient pollinator species, a widely reported factor contributing to the persistence and diversity of plant communities [51–53]. Similarly, Cunningham-Minnick *et al*. [21] found that, whilst flower visitation to crop plants was more frequent in plots with invasive flowers, the functional diversity of insect visitor species was reduced. For each focal species, we observed differences in overall visitation frequency between distant and invaded treatments, driven by changes in visitation by one main insect order in each case: Coleoptera for *C. edulis*, Diptera for *E. sesamoides*, and Hymenoptera for *O. moniliferum*. Therefore changes in visitation quality may be driven by these functional groups. A further factor that may affect seed production is that given the allelopathic properties of *Acacia* [25], below-ground chemical changes induced by close proximity to *Acacia* could impact plant fitness in nearby plots. Collecting seed from the ‘flower-removed’ plots would have allowed us to explore this possibility, by separating the vegetative and floral effects of *Acacia*.

While *O. moniliferum* and *E. sesamoides* had reduced visitor species richness and set fewer seeds at invaded plots, despite no significant change in the number of floral visits, *C. edulis* showed no significant change in seed production nor visitor species richness, despite receiving significantly fewer insect visits at invaded plots. The unchanged visitor species richness at invaded plots may have helped *C. edulis* to maintain seed set despite the reduced visitation it received. It is interesting that the change in insect richness and visitation between treatments was opposite for *C. edulis* to the other focal species. As both measures were significantly greater than for the other two species regardless of treatment, pollen and pollinator limitation may be reduced for this highly generalized species, buffering against heterospecific pollen transfer and/or changes in visitation caused by proximity to *Acacia* flowers. Additionally, species with short stigmas and small flowers, as *O*. *moniliferum* and *E*. *sesamoides* have, are more susceptible to heterospecific pollen transfer than larger species (59). To quantify the long-term implications of reduced seed production in native plants caused by *Acacia,* plant recruitment data are needed, as decreases in seed production do not necessarily lead to population-level changes if the plant species is not seed-limited [54]. Moreover, facultative self-pollinators such as *E. sesamoides* may be buffered against population-level changes caused by *Acacia* invasion.

## 5. Conclusion

Our research shows that stands of invasive *Acacia* can lead to significant changes in insect visitation and seed set in native co-flowering plant species *adjacent to* invaded stands, and this should be taken into account in the management of habitats adjacent to *Acacia*-invaded areas. A recent study detected changes in insect visitation to a flowering crop species up to 200 m away from an invaded area [21], indicating that such spillover effects can occur over considerable distances. Our study builds upon these findings in a natural habitat by using community-level network data, and evidence of reduced seed production in native plants. Given that research exploring the impacts of invasive plants tends to focus on native flora *within* invaded stands, this spillover effect presents an important avenue for further research in invasion ecology.

## Data Availability

All data and scripts are available via the Dryad Digital Repository [55]. Supplementary material is available online [56].
